# Case report: Successful management of a refractory double-expressor diffuse large B-cell lymphoma patient under the guidance of *in vitro* high-throughput drug sensitivity test

**DOI:** 10.3389/fonc.2022.1079890

**Published:** 2023-01-18

**Authors:** Lijie Xing, Hui Wang, Dan Liu, Qiang He, Zengjun Li

**Affiliations:** Department of Hematology, Shandong Cancer Hospital and Institute, Shandong First Medical University and Shandong Academy of Medical Sciences, Jinan, China

**Keywords:** DE-DLBCL, ATM, CD58, VTD, drug sensitivity screening

## Abstract

**Introduction:**

Double-expressor diffuse large B-cell lymphoma (DEL), harboring double expression of MYC and BCL2, has an inferior prognosis following standard first-line therapy with rituximab, cyclophosphamide, doxorubicin, vincristine, and prednisolone (R-CHOP). We initiated a clinical trial to treat newly diagnosed DEL with R-CHOP plus Bruton’s tyrosine kinase (BTK) inhibitor (BTKi) zanubrutinib (ZR-CHOP) and achieved a high complete response (CR) rate while four patients progressed during therapy, one of them carrying ATM and CD58 mutations. We applied an *in vitro* high-throughput drug sensitivity test for the prediction of clinical responses to different drugs in this patient.

**Case presentation:**

We report a 30-year-old female patient diagnosed with stage III (DEL), with ATM and CD58 mutations. The patient achieved partial response (PR) after two cycles of ZR-CHOP and remained PR after four cycles of ZR-CHOP, while the disease progressed after six cycles of ZR-CHOP. High-throughput drug screening using a panel of 117 compounds identified a range of therapies with efficacy for this patient. The primary tumor cells showed moderate sensitivity to bortezomib, thalidomide, and gemcitabine as a single agent and bortezomib, thalidomide, and dexamethasone (VTD) as a combined regimen. The patient was treated with two cycles of VTD regimen (bortezomib 1.3 mg/m^2^, d1, 4, 8, 11; thalidomide 100 mg, d1-21; dexamethasone 20 mg, d1, 2, 4, 5, 8, 9) and achieved PR with only a small lesion left. Another two cycles of VTD plus gemcitabine were then administered, and the patient achieved CR. Stem cells were mobilized, and autologous hematopoietic stem cell transplantation was carried out afterward. The patient remained CR for more than 3 months after transplantation.

**Conclusion:**

In this article, we present a first-line chemoresistant DEL patient with ATM and CD58 mutations who was treated successfully with VTD plus gemcitabine under the guidance of *in vitro* high-throughput drug sensitivity test.

## Introduction

Diffuse large B-cell lymphoma (DLBCL) is the most common subtype of non-Hodgkin’s lymphoma (NHL) with high heterogeneity. Approximately 30%~40% of DLBCL patients will develop relapsed or refractory disease, which is the major cause of mortality due to limited therapeutic options (1). Double-expressor DLBCL (DEL), harboring double expression of MYC and BCL2, represents nearly 1/3 of all DLBCL patients and shows a much poorer prognosis to rituximab plus cyclophosphamide, doxorubicin, vincristine, and prednisone (R-CHOP) treatment. The 5-year overall survival (OS) of DEL patients is only around 30%~40% with the standard regimen of R-CHOP (2), indicating there is an urgent clinical need for optimal treatment of these disease entities. We initiated a clinical trial to treat newly diagnosed DEL with R-CHOP plus Bruton’s tyrosine kinase (BTK) inhibitor (BTKi) zanubrutinib (ZR-CHOP) and obtained a promising complete response (CR) rate of 85.7% after six cycles of therapy (3), while four patients progressed during therapy, one of whom was carrying ATM and CD58 mutations, as reported here. We used extraordinary agents as the second line of therapy under the guidance of an *in vitro* high-throughput drug sensitivity test. The patient achieved a complete response, and consequently, autologous hematopoietic stem cell transplantation was completed.

## Case presentation

A 30-year-old female patient visited our hospital in May 2021, complaining of a nontender anterior neck mass rapidly increasing in size over the last month. The patient had no fever, night sweats, or weight loss. She lived a regular life and had no family history of malignant tumors. Physical examination revealed multiple swollen lymph nodes on the neck, which were firm, fixed, and nontender.

18F-FDG-PET showed the presence of fluorodeoxyglucose avid uptake in multiple parts, including several lymph nodes around the right carotid sheath with a maximum diameter of 3.4 cm and the standard uptake volume (SUVmax) of 33.3; an enlarged right tonsil with a SUVmax of 17.5; and a nodule in the soft tissue of upper segment of the left thigh with a length of 1.3 cm and a SUVmax of 6.3. No bone marrow infiltration was found by cytology, flow cytometry, and bone marrow biopsy.

The pathologic biopsy and immunohistochemistry (IHC) of neck lymph nodes revealed DLBCL, a nongerminal center B-cell-like (non-GCB) subtype, and overexpression of MYC (50%) and BCL2 (50%). The MYC, BCL2, and BCL6 rearrangements detected by fluorescence *in situ* hybridization (FISH) of the tumor tissue were negative.

Secondary gene detection was performed, and mutation analysis of the *DLBCL-43* gene was performed using capture-based next-generation sequencing (NGS) testing: ATM exon 27 p. C1366* missense mutation, abundance 38.1%; and CD58 exon p.I185fs frameshift mutation, abundance 16.4%. No mutations were detected in TP53.

Diagnosis of DLBCL (non-GCB subtype) was made based on her clinical presentation, morphology, and immunohistochemistry evaluation of lymph node specimens. She was in stage III, according to the Ann Arbor system. DLBCL (non-GCB subtype, DEL, stage III, group A, aaIPI 1) was confirmed by a multidisciplinary team composed of a pathologist, a radiologist, and an oncologist. The study was approved by the Institutional Review Board and carried out in accordance with the principles of the Declaration of Helsinki. Our study was approved by the Ethics Committee of the Shandong Cancer Hospital and Institute (Ethics No.: 2020-129-02). Informed consent was obtained from this patient.

The patient achieved a partial response (PR) after two cycles of ZR-CHOP (rituximab 375 mg/m^2^, d1; cyclophosphamide 750 mg/m^2^, d2; doxorubicin 50 mg/m^2^, d2; vincristine 1.4 mg/m^2^, d2 (to a maximum of 2 mg total dose); prednisolone 100 mg, d2-6; and zanubrutinib 160 mg, bid, d1-21). The patient remained PR after four cycles of ZR-CHOP, while the disease progressed after six cycles of ZR-CHOP. 18F-FDG-PET imaging demonstrated enlarged lymph nodes in the right neck area and an enlarged right tonsil with much higher FDG uptake (SUV_max_ = 32.6) compared to the liver (SUV_max_ = 3.0). A repeated biopsy of neck lymph nodes was performed and diagnosed with DLBCL, still with overexpression of MYC (50%~70%) and BCL2 (80%). The IHC showed CD20 (−), CD79a (little+), CD19 (−), PAX5 (+), CD3 (−), CD10 (+), MUM-1 (+), P53 (+, 80%, wild type), CD30 (−), CD5 (−), CyclinD1 (−), CD21 (−), Ki-67 (+, 80%), and EBER (−).

Due to the clinical situation and unfavorable prognosis, all therapeutic options were discussed with the patient. In addition to the current standard of second-line treatment options and clinical trials, the possibility of individual healing under the guidance of an *in vitro* high-throughput drug sensitivity test (DST) was discussed. The patient was informed in detail about the experimental nature of such treatment as well as the possible risks, and she actively agreed to receive the treatment under the guidance of DST.

**Figure 1:**
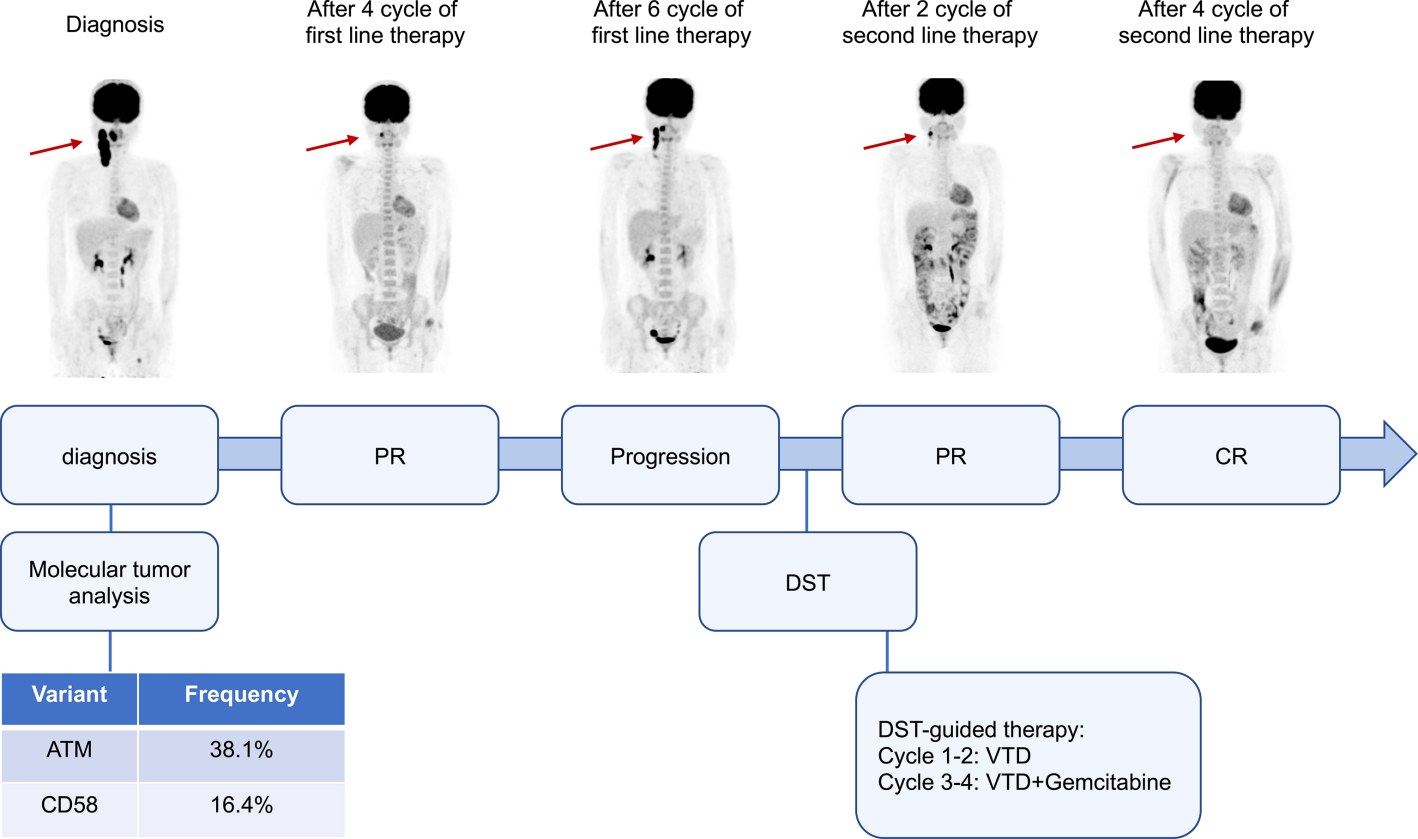
Clinical course and response images of the patient. PR, partial response; CR, complete response; DST, drug sensitivity test; VTD, bortezomib, thalidomide, and dexamethasone.

*In vitro* high-throughput drug sensitivity testing is a method for determining the sensitivity of tumor-fresh viable cells to agents (as many as several hundred) (4). This patient’s biopsy specimen of neck lymph nodes was used for DST. Primary cancer cells were obtained and amplified *in vitro*. Live cells were seeded and cultured in 384-well plates with drugs. Except for platinum drugs (oxaliplatin, cisplatin, carboplatin), all drugs were dissolved and diluted using dimethyl sulfoxide (DMSO). The concentrations were those used in clinical practice, which met the international drug standard. The control group was treated with DMSO. A dose (0.1 μl/well) was performed using a JANUS automated workstation. After incubation, cell viability was measured by the CellCounting-Lite 2.0 Luminescent Cell Viability Assay. The sensitivity of each treatment is listed in **Supplementary Table 1**. The primary tumor cells showed moderate sensitivity to bortezomib, thalidomide, gemcitabine as a single agent, and bortezomib, thalidomide, and dexamethasone (VTD) as a combined regimen. Gemcitabine is one of the routine drugs for relapsed/refractory DLBCL, while VTD is rarely reported for lymphoma treatment. The patient was treated with a VTD regimen, as that used in myeloma (bortezomib 1.3 mg/m^2^, d1, 4, 8, 11; thalidomide 100 mg, d1-21; dexamethasone 20 mg, d1, 2, 4, 5, 8, 9, 11, and 12). After two cycles of therapy, PR was obtained with only a small lesion left. Another two cycles of VTD plus gemcitabine were then administered, and the patient achieved a complete response (CR). No adverse events, such as peripheral neuropathy, pneumonia, liver and kidney function damage, and digestive tract discomfort, were detected during treatment. Stem cells were mobilized, and autologous hematopoietic stem cell transplantation was carried out afterward. The patients remained CR for more than 5 months after transplantation (**Figure 1**).

## Discussion

DLBCL is the most common non-Hodgkin lymphoma and accounts for about 40% of all NHL. R-CHOP is the standard regimen and cures about 60% of all patients, while the rest of the 40% are either refractory or relapsed after R-CHOP treatment. Throughout the past decades, the focus has been on how to improve the outcomes of DLBCL patients. However, few studies achieved positive results compared to those of R-CHOP. Given the high heterogeneity of DLBCL, accurately predicting outcomes and providing risk stratification or even personalized therapy can be one of the strategies.

DEL takes about 30% of DLBCL, and the 5-year PFS is only about 27% when treated with R-CHOP. However, studies of DEL are rare. In the CAVALLI study, seven out of eight (87.5%) double-hit DLBCL patients achieved CR, while no benefit for DEL patients compared with historical control (5). A phase II study of chidamide combined with an R-CHOP regimen in the treatment of elderly high-risk DLBCL patients showed that 100% of DE patients achieved CR, and the 2-year PFS rate and OS rate were 83% and 91%, respectively (6).

The BCR signaling pathway is highly activated in DEL patients, implying that a BTK inhibitor may be effective for DEL. In the Phoenix study, R-CHOP plus ibrutinib (IR-CHOP) had no benefit compared with R-CHOP plus placebo. Subgroup analysis showed IR-CHOP improved both PFS and OS markedly for younger patients (<60 years) with the double expression of c-MYC and BCL2. The benefit was counteracted in older patients due mainly to the issue of safety when combined with ibrutinib. Zanubrutinib is a more selective BTK inhibitor than ibrutinib. ZR-CHOP was initiated as a clinical study regimen in our center for DEL patients. A total of 89.3% patients (25/28) obtained CR after ZR-CHOP treatment, and only three progressed during therapy and follow-up (one patient was proved to be a false positive by PET-CT in the EHA report P1201, 85.7% CRR was reported). The case reported here is one of the three patients who progressed shortly after six cycles of ZR-CHOP.

Due to the high heterogeneity of DLBCL, it is difficult to accurately predict outcomes and provide individualized salvage therapies, both of which are essential for individualized cancer therapy (ICT). High-throughput DST is a personalized functional precision oncology approach that offers an assessment of additional possible treatments and combinations to identify effective therapeutic strategies for patients (4). For this patient, the primary tumor cells showed moderate sensitivity to bortezomib, thalidomide, and gemcitabine as a single agent, and VTD as a combined regimen. The result was confirmed by the clinical response to the treatment. Here, we will discuss the potential mechanisms underlying the efficacy, especially the effect of proteasome inhibitor (PI) on the tumor cells with ATM and CD58 mutations.

ATM is a protein kinase enzyme with a crucial role in the DNA repair system, acting as an intracellular sensor in response to DNA double-strand break (DSB) and then phosphorylating downstream proteins such as p53, chk2, and chk1, to initiate a cell-cycle arrest, apoptosis, and DNA repair (7). ATM alterations with putative pathogenic effects have been found in 13%–20% of DLBCL patients. The mutations of ATM were related to inferior PFS in localized DLBCL as well as GCB-DLBCL patients (8, 9).

ATM deficiency increases genomic instability by impairing DNA DSB repair as well as enhancing the dependence of cancer cells on other DNA repair mechanisms. Poly (ADP-ribose) polymerase-1 (PARP1) is another protein involved in DNA repair. PARP inhibitors have shown promising results in tumor cells defective in DNA damage repair (DDR) (10). In some studies, ATM loss showed that PARP inhibition was synthetically lethal, which is dependent on the tumor’s genetic background (11). Bortezomib (BTZ) was reported to impair the DNA homology-dependent repair (HDR), which is critical for the recovery of DNA DSB (12). In this patient, both DST and clinical results showed tumor cells were sensitive to BTZ treatment. As we know, when the amount of DNA damage exceeds the repair capacity, the damaged cells will be cleared through apoptosis. Therefore, we speculate that the DNA damage triggered by BTZ exceeds the repair capacity in ATM-deficient cells. Further preclinical studies are needed to reveal the new synthetic lethal effect of BTZ in the ATM mutation DLBCL. Indeed, inhibitors targeting other proteins in the DNA damage response are being developed for ATM-mutation cancers.

CD58 is the receptor for CD2, which is expressed on T cells and natural killer (NK) cells and is necessary for T-cell- and NK-cell-mediated cytotoxicity. CD58 mutations or loss occur in 21% of DLBCL patients, while the expression is deregulated in approximately 67% of DLBCL patients (12). BTZ can trigger specific antitumor immunity *via* immunogenic cell death (ICD), in which endogenous tumor cell proteins are recognized as damage-associated molecular patterns (DAMP) and activate cancer-specific immune responses (13). No studies show BTZ is effective in DLBCL with abnormal CD58. Further studies are needed to explore whether BTZ can overcome the immunodeficiency of patients with abnormal CD58 by inducing an ICD response.

This patient progressed after six cycles of ZR-CHOP chemotherapy, which may be related to ATM and CD58 mutations in addition to the poor response of patients with DEL. Proteasome inhibitors, such as bortezomib or carfilzomib, have shown encouraging efficacy in DLBCL but showed no benefit on PFS in the phase 3 study (14). However, in this case, the patient showed a good response to BTZ, which may be due to BTZ-induced DNA damage mutations leading to cell death in patients with ATM; BTZ overcomes the immunodeficiency of DLBCL caused by CD58 mutation by triggering the ICD response.

## Conclusion

Although gene mutations of ATM and CD58 increase molecular heterogeneity, they can be the potential therapeutic targets implicated in cancer therapy and clinical outcomes. To our knowledge, this case is the first chemoresistant DEL patient with ATM and CD58 mutations treated successfully with VTD plus gemcitabine, which provides new insights into the management of DLBCL. Prospective clinical trials are necessary to draw firm conclusions. Although there is still a long way to go in terms of curing DLBCL, optimal combinations of novel and traditional drugs will promote precision medicine in patients with DLBCL under the guidance of detailed genetic information.

## Data availability statement

The original contributions presented in the study are included in the article/**Supplementary Material**. Further inquiries can be directed to the corresponding author.

## Ethics statement

The studies involving human participants were reviewed and approved by Medical Ethical Committee of Shandong Cancer Hospital and Institute. The patients/participants provided their written informed consent to participate in this study. Written informed consent was obtained from the individual(s) for the publication of any potentially identifiable images or data included in this article.

## Author contributions

LX and ZL designed the study, performed treatments, collected and analyzed data, and wrote the manuscript. HW, DL, and QH collected data on clinical follow-up. All authors contributed to the article and approved the submitted version.
